# Myeloid sarcoma on the scalp of a patient with acute myeloid leukemia^☆^^☆☆^

**DOI:** 10.1016/j.abd.2019.09.001

**Published:** 2019-09-30

**Authors:** Débora Nogueira Muniz, Renata Cristina Vasconcellos, Letícia Ambrosano, Elisangela Samartin Pegas Pereira

**Affiliations:** a Medical Student, Pontifícia Universidade Católica de Campinas, Campinas, SP, Brazil; b Dermatology Outpatient Clinic, Pontifícia Universidade Católica de Campinas, Campinas, SP, Brazil

Dear Editor,

A 57-year-old female patient, Caucasian, with a previous diagnosis of hypertension, using losartan, and with acute myeloid leukemia for one year. She underwent chemotherapy with daunorubicin for three months and was in maintenance with transretinic acid 70 mg at the time of the consultation. She sought the dermatology service because of painful and itchy lesions on the scalp, which had arisen 20 days before and were preceded by local burning (Fig. 1). The proposed diagnostic hypotheses were cutaneous metastasis, cutaneous T-cell lymphoma, and pharmacodermia. An anatomopathological examination was performed, which demonstrated a dense diffuse infiltration of lymphocytic cells, atypical and large cells, four-to-five times the usual size of mature lymphocytes, with bizarre formats and convoluted nuclei in the superficial, intermediate, and deep dermis, with extension to the hypodermis, sparing the epidermis (Fig. 2). Immunohistochemical study of the lesion revealed diffuse positivity for CD43 in 80% of cell infiltrate, diffuse positivity for myeloperoxidase in more than 90% of cells, Ki67 positivity in about 30% of cells, and granzyme, alk1, CD3, CD20, and CD30 negativity (Fig. 3). Therefore, the diagnosis of myeloid sarcoma was established. At the end of the chemotherapy, she presented total remission of the hematological alterations; however, as she maintained lesions on the scalp, retreatment with radiotherapy was utilized. However, the lesions remained unchanged and the patient evolved with relapse of myeloid leukemia, with a new chemotherapy protocol initiated by the hematology team.

**Figure 1:**
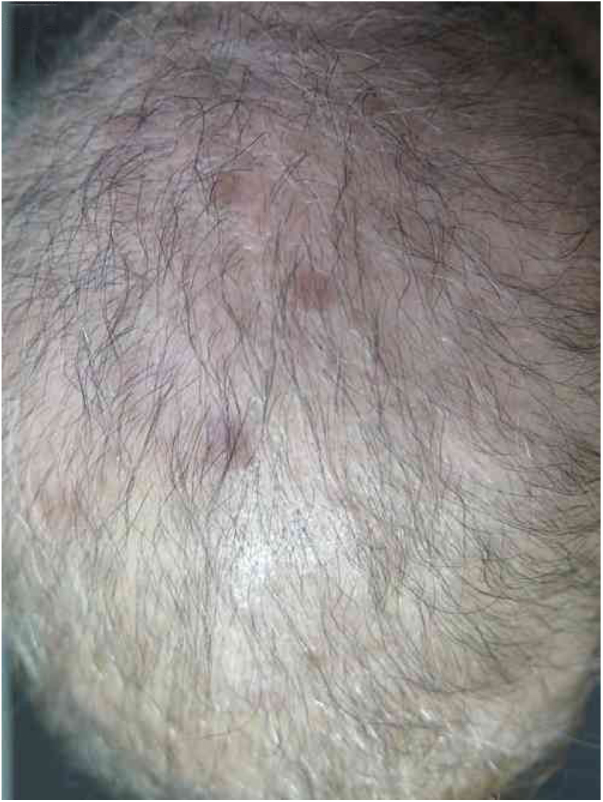
Scalp lesions.

**Figure 2:**
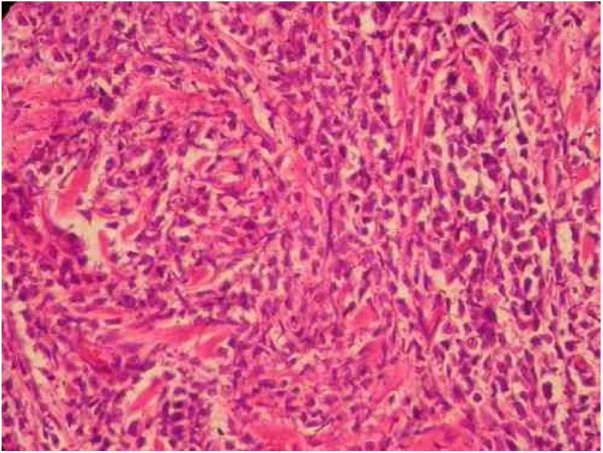
Intense diffuse lymphocytic infiltrate of atypical cells with extension to the hypodermis and sparing the epidermis. Cells are large in size, four-to-five times the usual size of mature lymphocytes, with bizarre formats and convoluted nuclei. 40× magnification. Coloration: hematoxylin and eosin.

**Figure 3:**
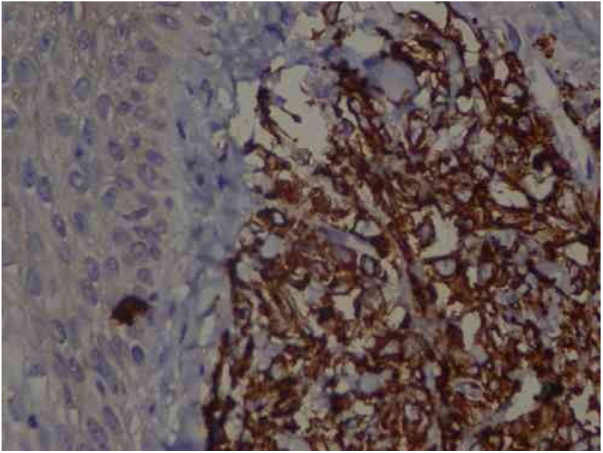
Immunohistochemistry: TCD4 diffuse positivity in 80% of lymphocytic infiltrate cells, focal positivity of 40% TCD3 cells, diffuse myeloperoxidase positivity in more than 90% of cells, TCD8 positivity in about 30% of cells, CD79a positivity in rare lymphoid cells of the dermis, Ki67 positivity in about 30% of the cells. 40× magnification.

Myeloid sarcoma, chloroma, or granulocytic sarcoma is defined by the World Health Organization as a solid tumor consisting of myeloblasts occurring in an anatomical site other than the bone marrow. Therefore, it should be remembered as a differential diagnosis of any atypical cellular infiltrate.^1^ In addition, it is a rare tumor, with an incidence of two in every 1,000,000 inhabitants, and is difficult to diagnose. This disease has great association with myeloproliferative diseases, especially with acute myeloid leukemia, and may be the first manifestation of the disease in 5% of cases. Cutaneous manifestations are more often seen as hardened, purplish, purpuric-based nodules, about 1–2.5 cm in diameter and more rarely as plaques, erythematous macules, blisters, and ulcers; they may present as an erythematous rash in a polymorphic pattern. It more commonly presents as a solitary lesion in places like soft tissues, bones, peritoneum, and lymph nodes. In the case reported, the lesion was present on the scalp, a rare site of disease occurrence.^2^ Studies have shown that the incidence of head and neck injuries is between 12% and 48%. The diagnosis is made through a biopsy with anatomopathological and immunohistochemical study, demonstrating infiltration by myeloblasts. The diagnosis by immunohistochemistry is mainly through positivity for the Leder stain, for lysozyme antigens, and for myeloperoxidase antigens. Bone marrow and myelogram biopsy should be performed to exclude other hematological malignancies. Many of the patients with myeloid sarcoma are misdiagnosed as having non-Hodgkin's lymphoma, Ewing's sarcoma, rhabdomyosarcoma, or neuroblastoma.^3^ Often, imaging tests, such as magnetic resonance imaging and computed tomography, should be used to elucidate the diagnosis when there is a central nervous system or musculoskeletal system impairment. Positron-emission tomography (PET) and computed tomography (CT) are used for radiotherapy planning and monitoring of therapeutic response. There are few studies regarding myeloid sarcoma, without consensus or protocols regarding treatment. There are also no prognostic studies. It is known that systemic and aggressive treatment is recommended for reducing the rate of progression to leukemia, using FLT 3 inhibitors, farnesyltransferase inhibitors, and histone deacetylase inhibitors.^4^, ^5^

An anatomopathological examination was performed (Fig. 2), which demonstrated a dense diffuse infiltration of lymphocytic cells, atypical and large cells, four-to-five times the usual size of mature lymphocytes, with bizarre formats and convoluted nuclei in the superficial, intermediate, and deep dermis, with extension to the hypodermis, sparing the epidermis. There was diffuse positivity for CD43 in 80% of lymphocytic infiltrate cells, diffuse myeloperoxidase positivity in more than 90% of cells, Ki67 positivity in about 30% of cells, and granzyme, alk1, CD3, CD20 and CD30 negativity. Therefore, the diagnosis of myeloid sarcoma was established. At the end of the chemotherapy, she presented total remission of the hematological alterations; however, as she maintained lesions on the scalp, she opted for retreatment with radiotherapy. However, the lesions remained unchanged and the patient evolved with relapse of myeloid leukemia, with a new chemotherapy protocol initiated by the hematology team.

## Funding

None declared.

## Author's contribution

Débora Nogueira Muniz: Elaboration and writing of the manuscript; obtaining, analyzing and interpreting the data; critical review of the literature.

Renata Cristina Vasconcellos: Conception and planning of the study; obtaining, analyzing and interpreting the data; critical review of the literature; critical review of the manuscript.

Letícia Ambrosano: Approval of the final version of the manuscript; effective participation in research orientation; intellectual participation in propaedeutic and/or therapeutic conduct of the cases studied.

Elisangela Samartin Pegas Pereira: Approval of the final version of the manuscript; conception and planning of the study; effective participation in research orientation; intellectual participation in propaedeutic and/or therapeutic conduct of the cases studied; critical review of the manuscript.

## Conflicts of interest

The authors declare no conflicts of interest.

## References

[1] Almond L.M., Charalampakis M., Ford S.J., Gourevitch D., Desai A. (2017). Myeloid sarcoma: presentation, diagnosis and treatment. Clin Lymphoma Myeloma Leuk.

[2] Yilmaz A.F., Saydam G., Sahin F., Baran Y. (2013). Granulocytic sarcoma: a systematic review. Am J Blood Res.

[3] Gupta A.J., Mandal S., Gupta R., Khurana N., Gulati A. (2017). Myeloid sarcoma presenting as nasal and orbital mass: an initial manifestation of an acute myeloid leukaemia. J Clin Diagn Res.

[4] Katagiri T., Ushiki T., Masuko M., Tanaka T., Miyakoshi S., Fuse K. (2017). Successful 5-azacytidine treatment of myeloid sarcoma and leukemia cutis associated with myelodysplastic syndrome: a case report and literature review. Medicine (Baltimore).

[5] Kono M., Allen P.K., Lin S.H., Wei X., Jeter M.D., Welsh J.W. (2017). Myeloid sarcoma on the scalp of a patient with acute myeloid leukemia. J Thorac Oncol.

